# Synergistic Anticancer Effects of Bleomycin and Hesperidin Combination on A549 Non-Small Cell Lung Cancer Cells: Antiproliferative, Apoptotic, Anti-Angiogenic, and Autophagic Insights

**DOI:** 10.3390/ph18020254

**Published:** 2025-02-14

**Authors:** Mehmet Kadir Erdogan, Guleser Ozer

**Affiliations:** 1 Department of Molecular Biology and Genetics, Faculty of Arts and Sciences, Bingol University, 12000 Bingol, Türkiye; 2 Department of Biology, Science Institute, Bingol University, 12000 Bingol, Türkiye

**Keywords:** lung cancer, bleomycin, hesperidin, apoptosis, autophagy, angiogenesis

## Abstract

**Background**: This study investigated the combined effects of hesperidin (Hesp), a natural flavonoid, with bleomycin (BL), a commonly used chemotherapy agent, on A549 human lung cancer cells. **Methods**: Key parameters assessed included cell viability, colony formation, and cell migration, alongside the expression of apoptotic and autophagic markers (p53, p21, Bax, cleaved PARP, and Beclin-1), VEGF levels, and caspase-3 activity. **Results**: The findings revealed that the Hesp + BL combination significantly amplified antiproliferative, apoptotic, anti-angiogenic, and autophagic effects compared to either treatment alone. The combination therapy effectively inhibited colony formation and cell migration while markedly reducing VEGF levels, indicating strong anti-angiogenic properties. Apoptotic markers such as p53, p21, Bax, and cPARP were significantly upregulated, with caspase-3 activity confirming robust apoptosis induction. Furthermore, autophagy was notably enhanced, as reflected by increased Beclin-1 expression. **Conclusions**: Synergistic interactions between Hesp and BL, validated through combination index analysis, underscore the therapeutic potential of this combination. These findings underscore the therapeutic potential of the Hesp + BL combination as a promising strategy for lung cancer treatment, meriting further exploration in diverse lung cancer cell lines to validate and expand its applicability in developing novel therapeutic approaches.

## 1. Introduction

Cancer is defined by the abnormal and uncontrolled growth of cells, with the potential to invade surrounding tissues and metastasize throughout the body [1]. Key factors in cancer progression include the inactivation of tumor suppressor genes, overexpression of oncogenes, genomic instability, genetic and epigenetic mutations, alterations in the tumor microenvironment, dysregulated intracellular signaling pathways, and defective apoptosis [2]. In 2022, approximately 20 million new cancer cases were reported globally, alongside 9.7 million cancer-related deaths. Lung cancer remains the most diagnosed type, accounting for 12.4% of all cases (around 2.5 million) and 18.7% of cancer deaths (about 1.8 million) [3]. While around 90% of lung cancer cases are linked to smoking and tobacco use, environmental and genetic factors, including exposure to radon gas, asbestos, air pollution, chronic infections, and hereditary predispositions, also contribute to its development [4]. Current treatments for lung cancer include surgery, radiation therapy, chemotherapy, and targeted therapies, with recommendations depending on the cancer’s type and stage [5]. Despite advancements in diagnosis and treatment, lung cancer prognosis remains poor, and responses to conventional therapies are often limited, except in cases of localized tumors. A deeper understanding of lung cancer biology may lead to more effective and targeted treatments [4].

Chemotherapy and radiotherapy, while commonly used, are associated with significant side effects, high costs, and limited effectiveness due to toxicities [6]. This underscores the urgent need for safer and more effective chemotherapeutic agents. Phytochemicals, natural bioactive compounds derived from plants, have emerged as promising alternatives. Several phytochemicals, such as camptothecin, vincristine, and paclitaxel, have been successfully developed for their potent anticancer properties and are now widely used in clinical practice [6].

**Figure 1:**
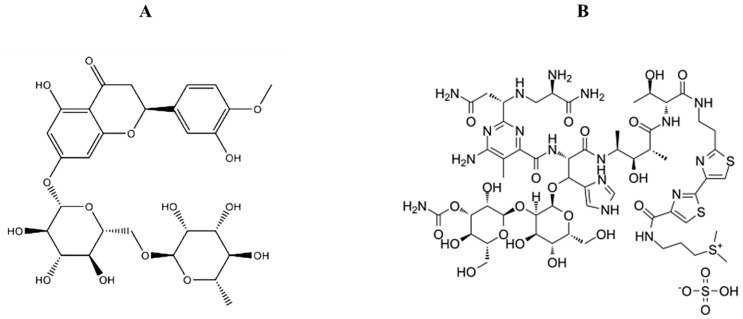
Chemical structures of Hesp (**A**) and BL (**B**).

BL is a water-soluble glycopeptide antibiotic with a molecular weight of approximately 1500 Da (Figure 1B). The form commonly administered, BL sulfate, is used as a chemotherapeutic agent in cancer treatment [7]. However, BL is associated with serious side effects, including pulmonary toxicity, nephrotoxicity, and gastrointestinal distress. Patients undergoing BL therapy often experience significant physical and psychological distress, negatively impacting their quality of life. Thus, developing therapeutic strategies that reduce BL’s toxicity while enhancing its efficacy is crucial for improving patient outcomes [7].

In developed countries, nearly half of all cancer patients use complementary and alternative therapies alongside conventional treatments [8]. Among these, plant-based treatments have been widely used for various diseases, including cancer. Plants have historically been used for multiple purposes, from nutrition and cosmetics to therapeutic applications, and have played a key role in the development of pharmaceutical products [8]. Recently, natural phytochemicals have gained attention for their accessibility, affordability, low toxicity, and minimal side effects, offering potential solutions to the limitations of conventional therapies [5,8].

Natural compounds are increasingly being explored for their therapeutic potential in drug development due to their structural diversity and promising clinical outcomes. Many of these compounds exhibit anticancer and antioxidant properties, making them valuable candidates for novel cancer therapies [5]. Certain plant-based agents are known to influence cancer-related signaling pathways, regulate gene expression, and modulate cell cycle and apoptosis. Proteomic studies have shown that some plant-derived compounds affect proteins involved in these pathways, inducing apoptosis in cancer cells [9].

Citrus fruits, such as oranges, lemons, and limes, are recognized for their health benefits and chemopreventive properties. One of the key bioactive compounds in these fruits is hesperidin (Hesp), a flavonoid derived from hesperetin. Hesp (3′,5,7-trihydroxy-4′-methoxyflavanone-7-6-O-α-L-rhamnosyl-D-glucose) is a natural flavonoid abundant in various fruits and vegetables [10] (Figure 1A). A wide range of pharmacological activities of Hesp has been reported, including potent antioxidant, anti-inflammatory, cardioprotective, neuroprotective, and anticancer properties [11,12,13,14]. Hesp prevents malignant transformation and cancer progression by targeting molecular mechanisms involved in tumor cell survival, proliferation, and apoptosis through upregulation of Bax and caspase-3, downregulation of Bcl-2, and stimulation of NF-κB and AKT/mTOR signaling pathways [12,13]. Two different studies have shown that Hesp exhibits therapeutic effects against BL-induced pulmonary fibrosis in male Sprague Dawley rats by regulating inflammatory markers through inhibition of the TGF-β1/Smad3/AMPK and IκBα/NF-κB pathways [15], as well as by regulating lung inflammatory markers TNF-α and IL-1β, and inducing Nrf-2 [16]. In a recent study using MRC-5 lung fibroblast cells, Hesp was reported to prevent lung fibroblast senescence in vitro via downregulation of the expression of senescence marker proteins (p53, p21 and p16) and inhibition of the IL6/STAT3 signaling pathway, and also to alleviate BL-induced pulmonary fibrosis in vivo [17].

The objective of this study is to investigate the synergistic effects of Hesp and BL on cell proliferation, apoptosis, autophagy, and angiogenesis in A549 non-small cell lung cancer cells. A549 cells were cultured and treated, after which the antiproliferative, apoptotic, autophagic, and anti-angiogenic effects of the treatments were evaluated. Additionally, key protein expression levels involved in apoptotic and autophagic pathways were analyzed. This study aims to elucidate the molecular mechanisms of this combined therapy and contribute to the development of new, targeted cancer treatments with improved selectivity and efficacy.

## 2. Results

### 2.1. Hesp, BL and Their Combination Reduces Viability of A549 Non-Small Cell Lung Cancer Cells in a Dose-Dependent Manner

**Figure 2:**
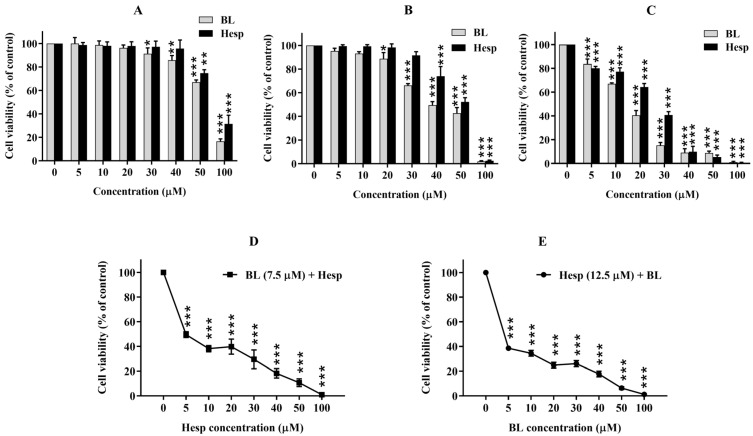
The cell viability findings in A549 cells after 24 h (**A**), 48 h (**B**) and 72 h (**C**) treatment with Hesp and BL. In addition, the effect of combination treatments with increasing doses of Hesp while keeping the BL concentration constant (**D**), and with increasing doses of BL keeping the Hesp concentration constant (**E**), on cell viability was also determined. Cell viability was determined by WST-1 assay. Results represent mean ± standard deviation (SD). One-way ANOVA and Tukey’s multiple comparison test were used in statistical analyses. * *p* < 0.05, ** *p* < 0.01, *** *p* < 0.001, compared to untreated cells.

The antiproliferative effects of Hesp, BL, and their combination on A549 lung cancer cells were evaluated using the WST-1 cell viability assay. A reduction in A549 cell viability was observed after 24 h of treatment with both Hesp and BL, particularly at higher concentrations (Figure 2A). At a concentration of 100 µM, cell viability decreased by 68.53% for Hesp-treated cells and 83.35% for BL-treated cells compared to untreated controls. After 24 h of treatment, the IC_50_ values were calculated as 82.85 ± 2.91 µM for Hesp and 70.12 ± 0.91 µM for BL.

After 48 h of treatment, a significant reduction in cell viability was noted at concentrations of 40 µM and above for both Hesp and BL (Figure 2B). At 100 µM, the proliferation of Hesp-treated cells was inhibited by 97.56%, while BL-treated cells showed a 98.02% inhibition compared to the control group. The IC_50_ values after 48 h were 55.43 ± 3.14 µM for Hesp and 41.87 ± 1.53 µM for BL.

The antiproliferative effects of Hesp and BL were even more pronounced after 72 h of treatment, with cell viability decreasing in a dose-dependent manner (Figure 2C). The IC_50_ values at 72 h were determined to be 24.27 ± 1.41 µM for Hesp and 14.95 ± 1.28 µM for BL, indicating a much greater efficacy compared to the 24 h and 48 h treatments.

### 2.2. Combination of Hesp and BL Exhibits Synergistic Interaction

Concentrations of 12.5 µM for Hesp and 7.5 µM for BL were used to determine the combination index for the Hesp + BL treatment, which were approximately half of the IC_50_ values obtained after 72 h of exposure. The absorbance values of cells treated with only 12.5 µM Hesp or 7.5 µM BL were normalized to 100% viability for calculation purposes. A549 cells were treated for 72 h with increasing concentrations of BL, while the Hesp concentration remained constant at 12.5 µM. This combination was found to decrease cell viability in a dose-dependent manner, in line with the increasing BL concentration (Figure 2D). In the presence of 12.5 µM Hesp, the IC_50_ value of BL was determined to be 7.58 ± 0.32 µM. Similarly, when A549 cells were treated with increasing concentrations of Hesp combined with a constant 7.5 µM BL for 72 h, the inhibition of cell proliferation increased in a dose-dependent manner (Figure 2E). Under these conditions, the IC_50_ value of Hesp was found to be 9.95 ± 0.80 µM.

Combining a therapeutic drug with a herbal agent can enhance treatment effectiveness by targeting different cellular mechanisms or pathways [18]. For a combination therapy to be considered synergistic, several beneficial outcomes should be observed, such as increased therapeutic efficacy, maintaining or improving the same efficacy at lower doses, reducing or preventing drug resistance, and achieving selective synergy against a specific target or mechanism. These synergistic effects are the foundation for the widespread use of drug combinations, significantly improving treatment outcomes for serious diseases like cancer [18]. Based on the formula provided in the Section 4 [19], the combination index (CI) for the Hesp + BL treatment was calculated to be 0.92. Since this value is less than 1 (CI < 1), it indicates a synergistic interaction between Hesp and BL.

### 2.3. Colony Survival Is Suppressed in the Presence of Hesp, BL or Their Combinations

**Figure 3:**
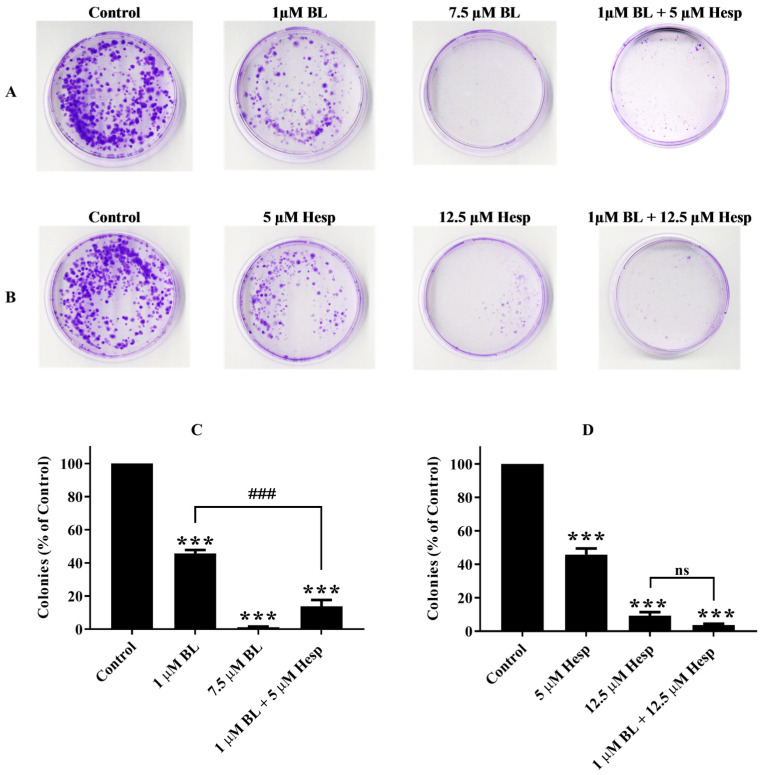
Clonogenic assay findings. (**A**,**B**). A549 cells treated with different concentrations of Hesp, BL or the combination of BL + Hesp for 72 h. (**C**,**D**). The colony numbers formed were normalized with the control groups, and the findings were calculated quantitatively in terms of % colonies. Data are shown as mean ± SD. One-way ANOVA and Tukey’s multiple comparison test were used in statistical analyses. *** *p* < 0.001, compared to control group. ### *p* < 0.001, ns: *p* > 0.05, compared to BL-treated cells.

The colony formation assay is an in vitro survival test that measures the ability of a single cell to grow into a colony, which is defined as a cluster of at least 50 cells. This assay evaluates the capacity of individual cells within a population to undergo “unlimited” division. It is often used to assess the effects of cytotoxic agents on cell proliferation, as only a subset of transplanted cells possess the potential for colony formation [20]. The suppressive effects of 72 h treatments with Hesp, BL, and their combination on the colony-forming ability of A549 cells were evaluated using the colony formation assay (Figure 3A,B).

As shown in Figure 3C,D, colony formation was reduced by 54.20% in cells treated with 12.5 µM Hesp and by 54.19% in cells treated with 1 µM BL, compared to untreated controls (*p* < 0.001). A549 cells treated with the combinations of 1 µM BL + 5 µM Hesp and 1 µM BL + 12.5 µM Hesp exhibited a reduction in colony formation by 86.17% and 96.32%, respectively, relative to untreated cells (*p* < 0.001) (Figure 3C,D). The combination of 1 µM BL + 5 µM Hesp significantly suppressed colony formation compared to treatment with 1 µM BL alone (*p* < 0.001). However, the combination of 1 µM BL + 12.5 µM Hesp did not show a significant difference in colony formation compared to the 12.5 µM Hesp treatment alone (*p* > 0.05, not significant).

### 2.4. Hesp, BL and Their Combination Inhibits the Migration of A549 Cells

**Figure 4:**
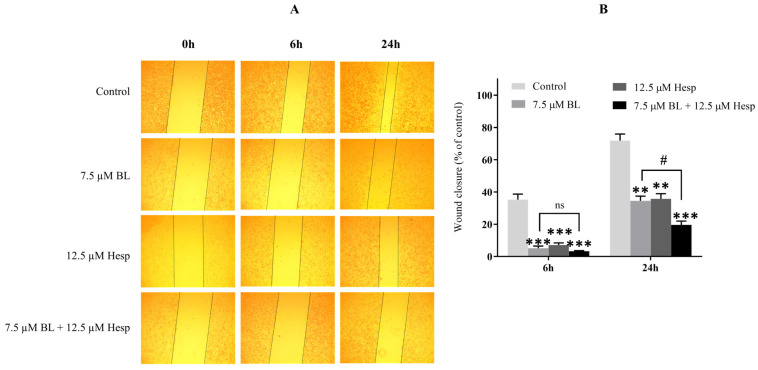
Scratch wound-healing assay (cell migration). (**A**). A549 cells were treated with Hesp, BL or the combination of BL + Hesp and visualized under a microscope (×10 magnification) at 6 and 24 h after wounding. Open areas define areas where cells are missing (wound area, ImageJ). (**B**). Percent wound closure values are given as mean ± SD. One-way ANOVA and Tukey’s multiple comparison test were used in statistical analyses. ** *p* < 0.01, *** *p* < 0.001 compared to control group. # *p* < 0.05, ns: *p* > 0.05 compared to BL-treated cells.

The wound-healing assay is one of the widely used techniques developed to study in vitro cell migration, simulating the process that occurs during wound healing in vivo. The primary steps involve creating a “wound” in a cell monolayer, capturing images at the start and at regular intervals as the cells migrate to close the wound, and then comparing these images to assess the migration rate. This method is particularly useful for studying the effects of cell–matrix and cell–cell interactions on migration [21]. In this study, wound-healing assays were conducted to evaluate the migration rate of cells treated with Hesp, BL, and their combination (Figure 4A).

Each group was normalized to its respective 0 h values. As shown in Figure 4B, the wound closure after 6 h was significantly delayed in cells treated with 7.5 µM BL, 12.5 µM Hesp, and the combination of 7.5 µM BL + 12.5 µM Hesp compared to the control group (*p* < 0.001). However, there was no statistically significant difference in migration rates between the combination treatment and cells treated with 7.5 µM BL alone (*p* > 0.05, ns). After 24 h, the migration rate was significantly reduced in cells treated with 7.5 µM BL and 12.5 µM Hesp compared to untreated cells (*p* < 0.01). In the cells treated with the combination therapy, the reduction in migration rate was even more pronounced than in the control group (*p* < 0.001). Additionally, the difference in migration rates between the combination treatment and 7.5 µM BL alone was statistically significant (*p* < 0.05) (Figure 4B). These findings suggest that the Hesp + BL combination significantly slows the wound-healing process, indicating an inhibition of cell migration.

### 2.5. Hesp + BL Combination Treatment Reduces VEGF Levels

**Figure 5:**
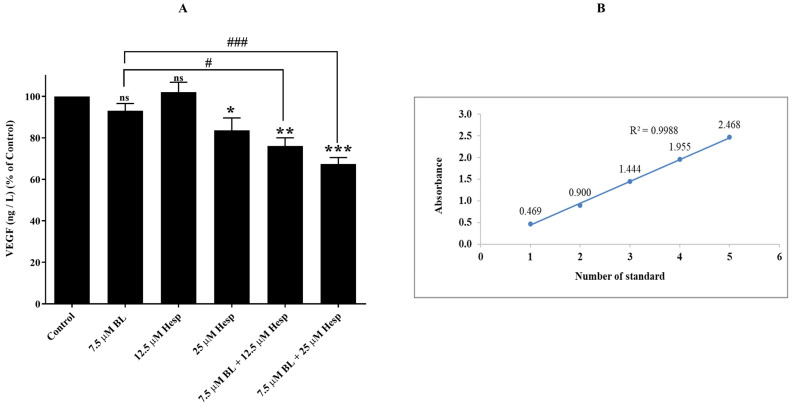
(**A**). Anti-angiogenic effect of Hesp, BL and combined treatments in A549 cells. (**B**). The findings were normalized with the graph created using the standard compounds in the kit content and percent VEGF amount values are given as mean ± SD. One-way ANOVA and Tukey’s multiple comparison test were used in statistical analyses. * *p* < 0.05, ** *p* < 0.01, *** *p* < 0.001, ns: *p* > 0.05 compared to control cells. # *p* < 0.05, ### *p* < 0.001 compared to BL-treated cells.

VEGF is a potent inducer of angiogenesis and lymphangiogenesis, acting as a highly specific mitogen for endothelial cells. Its signal transduction occurs through binding to tyrosine kinase receptors, leading to endothelial cell proliferation, migration, and the formation of new blood vessels. The role of VEGF is closely linked to angiogenesis, a critical step in tumor development [22]. Absorbance values for the standards are shown in Figure 5B. These values were used for normalization to determine the VEGF levels in treated cells.

As illustrated in Figure 5A, there was no significant change in VEGF levels in cells treated with 7.5 µM BL and 12.5 µM Hesp compared to the control group (*p* > 0.05, not significant). However, treatment with 25 µM Hesp resulted in a 16.41% reduction in VEGF levels (*p* < 0.05). Cells treated with the combination of 7.5 µM BL + 12.5 µM Hesp and 7.5 µM BL + 25 µM Hesp showed a VEGF decrease of 23.94% and 32.61%, respectively (*p* < 0.01 and *p* < 0.001). These findings suggest that the combination therapy significantly reduces VEGF levels compared to BL alone, indicating a strong anti-angiogenic effect.

### 2.6. Cellular Vacuolization and Beclin-1 Protein Expression Levels Indicate the Autophagic Effect of Combined Treatment

**Figure 6:**
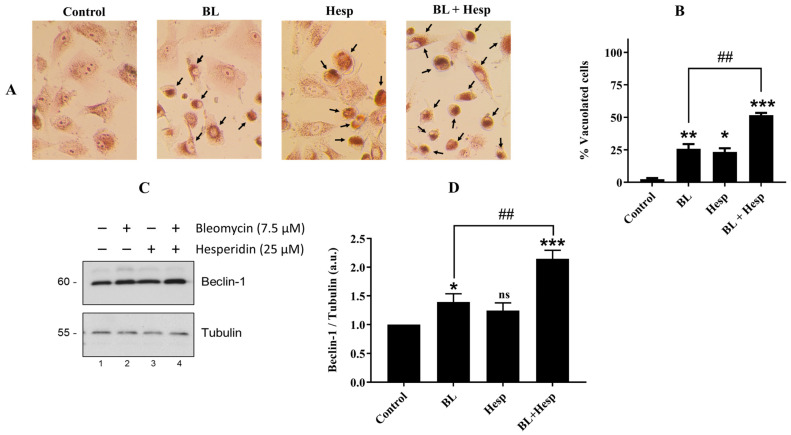
Autophagic effect of Hesp, BL and Hesp + BL treatments in A549 cells. (**A**). After treatments, cells were stained with neutral red to determine the formation of autophagic vacuoles. Black arrows indicate autophagic vacuoles in cells. (**B**). After counting the cells that formed autophagic vacuoles and the normal cells under a microscope at ×40 magnification, the findings were presented graphically to show the quantitative results of the % vacuolated cells. (**C**). Beclin-1 protein levels determined by Western blot analysis. (**D**). Protein expression levels were normalized with tubulin and expressed as fold change. Data are given as mean ± SD. One-way ANOVA and Tukey’s multiple comparison test were used in statistical analyses. * *p* < 0.05, ** *p* < 0.01, *** *p* < 0.001, ns: *p* > 0.05, compared to control group. ## *p* < 0.01, compared to BL-treated cells.

The presence of acidic vacuoles, a hallmark of autophagy, has been linked to apoptosis following autophagy [23]. The accumulation of lysosomal acidic vacuoles was significantly increased in cells treated with 7.5 µM BL (*p* < 0.01), 12.5 µM Hesp (*p* < 0.05), and the combination of these two concentrations (*p* < 0.001), as shown in Figure 6A,B. Notably, morphological observations revealed a greater accumulation of acidic vacuoles in cells treated with the combination therapy compared to the BL group alone (*p* < 0.01). These findings suggest that autophagy-induced cell death is strongly associated with the combined treatment of Hesp with BL.

Beclin-1, a key autophagy-related protein, also functions as a tumor suppressor in mammalian cells. Beclin-1 contains a conserved BH3 domain that binds to Bcl-2 or Bcl-XL, suggesting that Beclin-1 may play a direct role in initiating apoptotic signaling by interacting with Bcl-2 family proteins. Bcl-2, when bound to Beclin-1, inhibits autophagy, while the release of Beclin-1 from the Beclin-1–Bcl-2 or Beclin-1–Bcl-XL complexes induces autophagy [24]. Protein expression levels of Beclin-1, in A549 cells treated with BL, Hesp and their combinations were also analyzed by Western blot. As shown in Figure 6C,D, the combination of Hesp and BL increased Beclin-1 levels by 2.15-fold compared to the control group (*p* < 0.001), and this increase was significantly higher compared to treatment with BL alone (*p* < 0.01), suggesting that the BL + Hesp combination promotes autophagy in A549 cells, likely in connection with apoptotic activity.

### 2.7. Combination of Hesp and BL Induces Apoptosis in A549 Non-Small Cell Lung Cancer Cells

**Figure 7:**
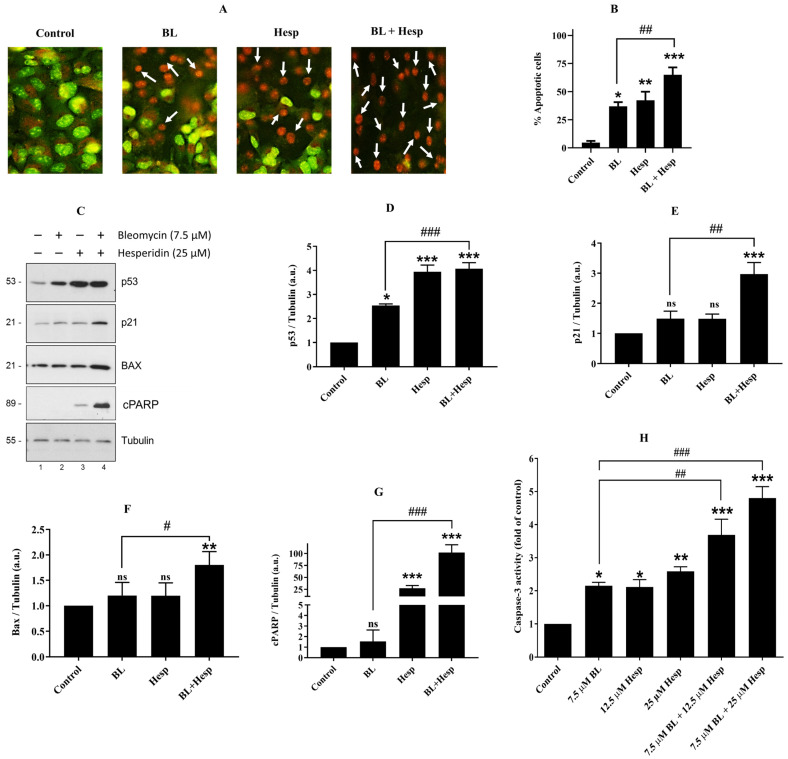
The apoptotic activity of Hesp, BL and Hesp + BL treatments on A549 cells. (**A**). AO/EB dual staining was used to visualize live and apoptotic cells. White arrows indicate apoptotic cells. (**B**). After counting under the microscope at ×40 magnifications, graphs showing the quantitative results of % apoptotic cells were drawn. (**C**). Western blot findings. Following treatments, lysates obtained from cells were subjected to SDS-PAGE electrophoresis, band images in the gel were transferred to nitrocellulose membrane, and the expression levels of p53 (**D**), p21 (**E**), Bax (**F**), and cPARP (**G**) were quantitatively determined. Tubulin was used for normalization. (**H**). Colorimetric determination of caspase-3 was performed by an ELISA-based analysis, and the results are expressed quantitatively. Data are given as mean ± SD. One-way ANOVA and Tukey’s multiple comparison test were used in statistical analyses. * *p* < 0.05, ** *p* < 0.01, *** *p* < 0.001, ns: *p* > 0.05, compared to control. # *p* < 0.05, ## *p* < 0.01, ### *p* < 0.001, compared to BL-treated cells.

Apoptosis is a programmed cell death process that occurs as part of the normal cellular life cycle, while the ability to evade apoptosis is a hallmark of cancer cells. During apoptosis, DNA undergoes internucleosomal fragmentation, chromatin condenses, the nucleus fragments, and the cell membrane forms blebs, leading to cell shrinkage and the formation of apoptotic bodies that contain the cell’s contents. As shown in Figure 7A,B, treatment with Hesp, BL, and combination significantly reduced the number of normal cells compared to the control group, while the number of apoptotic cells increased significantly (*p* < 0.05, *p* < 0.01, and *p* < 0.001, respectively). In particular, when the BL + Hesp (combined)-treated group was compared with BL-treated cells, there was a notable decrease in the normal cell count alongside a significant increase in apoptotic cells (*p* < 0.01) (Figure 7B).

The levels of apoptotic proteins in A549 lung cancer cells treated with BL, Hesp, and their combination were analyzed with Western blot (Figure 7C). The resulting band intensities were normalized to tubulin, and changes in protein levels were expressed as fold increases or decreases. As seen in Figure 7D, p53 expression increased by 2.54-fold in cells treated with 7.5 μM BL compared to the control (*p* < 0.05). In contrast, cells treated with 25 μM Hesp and the combination of 7.5 μM BL + 25 μM Hesp exhibited 3.94- and 4.07-fold increases in p53 expression, respectively (*p* < 0.001). The combination treatment significantly elevated p53 levels compared to BL treatment alone (*p* < 0.001). As a tumor suppressor, p53 regulates cell cycle arrest and apoptosis in response to genotoxic stress and DNA damage [25]. It is also recognized as a transcription factor that modulates the expression of genes involved in proliferation, cell cycle regulation, and apoptosis [26]. Studies have demonstrated that p53 inhibits cancer cell proliferation by halting the cell cycle and inducing apoptosis through activation of tumor suppressor genes such as DR5, p21, caspase-8, and Bax [27]. In this study, increased p53 expression observed in A549 cells treated with the combination of BL + Hesp revealed that apoptosis was triggered by the combined treatment.

p21, a cyclin-dependent kinase (CDK) inhibitor, plays a critical role in promoting cell cycle arrest in response to various stimuli. The inhibitory effect of p21 on cell cycle progression is closely linked to its nuclear localization and can be induced through both p53-dependent and p53-independent mechanisms. In addition to regulating the cell cycle, p21 is involved in transcriptional modulation. It is activated after DNA damage, temporarily arresting cells at the G1 and G2 checkpoints, providing time for DNA repair [28]. As shown in Figure 7E, p21 levels increased 2.97-fold in BL + Hesp-treated cells compared with control (*p* < 0.001), and this increase was also significant compared to BL treatment alone (*p* < 0.01), suggesting that the combination treatment acts by inducing cell cycle arrest in response to DNA damage.

Bax, a pro-apoptotic member of the Bcl-2 family, permeabilizes the mitochondrial outer membrane to release cytochrome c, thereby activating the caspase cascade. Inactivating mutations in Bax are common in many cancers, including lung cancer, and lead to unchecked tumor growth [29]. In this study, no significant difference in Bax expression was observed between untreated cells and cells treated with BL or Hesp alone (*p* > 0.05, ns). However, the combination of 7.5 μM BL + 25 μM Hesp increased Bax expression by 1.81-fold compared to the control group (*p* < 0.01), and significantly higher Bax expression was detected in the combination treatment group compared to BL alone (*p* < 0.05) (Figure 7F). These findings suggest that the BL + Hesp combination directs A549 cells to apoptosis through the mediation of the pro-apoptotic Bax protein.

cPARP is a hallmark of apoptosis, as it is cleaved by activated caspases. To further validate that BL, Hesp, and their combination induce apoptosis in A549 cells via p53-regulated pathways, we examined the abundance of cPARP. Densitometric analysis revealed no significant change in cPARP levels in BL-treated cells (*p* > 0.05, ns), but Hesp- and BL + Hesp-treated cells exhibited a 27.2- and 102.1-fold increase in cPARP levels, respectively (Figure 7G). These results indicated that the combination treatment significantly enhances caspase-mediated DNA fragmentation and apoptosis (Figure 7C, fourth panel, lanes 1–4).

Caspases are key mediators of apoptosis, specifically type-I programmed cell death, with caspase-3 being one of the most frequently activated enzymes. Caspase-3 plays a crucial role in cleaving various cellular proteins following mitochondrial membrane disruption. Unlike cytoplasmic caspases, which contribute to mitochondrial membrane permeabilization, caspase-3 activation depends on the breakdown of the mitochondrial membrane and the subsequent release of the intermembrane protein, cytochrome c [30]. The effects of the treatments on caspase-3 levels were measured colorimetrically (Figure 7H). In cells treated with 7.5 µM BL, 12.5 µM Hesp, and 25 µM Hesp, caspase-3 levels were found to be 2.15-fold (*p* < 0.05), 2.11-fold (*p* < 0.05), and 2.59-fold (*p* < 0.01) higher, respectively, compared to the control. In the combined treatment groups, caspase-3 levels were 3.69-fold and 4.80-fold higher for the 7.5 µM BL + 12.5 µM Hesp and 7.5 µM BL + 25 µM Hesp treatments, respectively, compared to the untreated cells (*p* < 0.001). The combined treatment significantly increased caspase-3 levels compared to BL-treated cells (*p* < 0.01).

## 3. Discussion

Lung cancer, characterized by genetic and epigenetic changes that drive clonal expansion and ultimately lead to invasive cancer, remains a significant clinical challenge due to its heterogeneity and late-stage diagnosis [4]. Non-small cell lung carcinoma (NSCLC), the most prevalent form, accounts for approximately 85% of cases and includes diverse subtypes such as squamous cell carcinoma, adenocarcinoma, and large cell carcinoma. Despite advances in surgery, chemotherapy, and radiation therapy, the 5-year survival rate for NSCLC remains low, particularly in advanced-stage diagnoses [31]. This highlights the urgent need for more effective and targeted therapies that can overcome resistance and reduce toxicity. In this study, we investigated the combined effects of BL and Hesp on A549 NSCLC cells, focusing on the induction of apoptosis, autophagy, and angiogenesis inhibition. Our results demonstrate a clear synergistic effect of BL and Hesp in promoting cell death through both apoptotic and autophagic pathways, suggesting that Hesp enhances the therapeutic potential of BL, a chemotherapy agent widely used in the treatment of various cancers [32].

The term ‘regulated cell death’ (RCD) is used to refer to cell death that can be influenced by certain genetic or pharmacological interventions [33]. Apoptosis, one of the subtypes of RCD, is an essential defense mechanism against cancer progression, enabling the removal of damaged or mutated cells that could otherwise lead to malignancy. However, many cancer cells, including those in lung cancer, develop strategies to evade apoptosis, resulting in uncontrolled proliferation and tumor growth [1]. Our study revealed that the combination of Hesp with BL significantly enhanced the expression of critical apoptotic markers, including p53, p21, Bax, and cPARP, the latter being a hallmark of caspase-mediated apoptosis. Notably, p53, a tumor suppressor pivotal in DNA repair and cell cycle regulation, was upregulated in response to the combination therapy compared to BL alone, indicating that Hesp augments BL’s ability to activate the intrinsic mitochondrial apoptotic pathway. This was further supported by the observed increases in Bax expression, and cPARP levels, demonstrating robust apoptotic activation. In addition to apoptosis, autophagy, a cellular recycling process triggered under stress conditions, plays a dual role in cancer, either promoting survival or inducing autophagic cell death when cellular stress becomes excessive [25]. Our findings indicated that the combination of BL and Hesp significantly increased Beclin-1 expression, a central regulator of autophagy, suggesting that autophagy was induced in A549 cells. This induction implies that autophagy contributed to the cell death process, potentially interacting with apoptotic pathways to amplify the therapeutic effects. These results align with previous studies showing that Hesp can simultaneously modulate apoptosis and autophagy, thereby enhancing the efficacy of conventional cancer treatments. Together, the dual activation of these pathways by the BL-Hesp combination underscores its potential as a synergistic strategy for lung cancer therapy, offering a promising avenue for further research and therapeutic development.

The synergistic interaction between BL and Hesp can be attributed to their complementary mechanisms of action. BL induces DNA damage by binding to DNA and creating strand breaks, primarily at GC-rich regions [34]. Hesp, a natural flavonoid with antioxidant, anti-inflammatory, and anticancer properties, modulates several signaling pathways, including those related to apoptosis and autophagy. Studies have demonstrated that Hesp can induce apoptosis through the mitochondrial pathway by increasing reactive oxygen species (ROS) levels and promoting the activation of pro-apoptotic proteins such as Bax [35]. In our study, the combination of BL and Hesp appeared to enhance DNA damage, leading to a greater activation of apoptotic and autophagic processes compared to BL alone. This suggests that Hesp amplifies BL’s DNA-damaging effects while simultaneously triggering additional pro-death pathways.

Angiogenesis, the formation of new blood vessels, is crucial for tumor growth and metastasis. Vascular endothelial growth factor (VEGF) is a key mediator of angiogenesis in cancer, and its inhibition is a common therapeutic target [36]. Our results showed that the BL + Hesp combination significantly reduced VEGF levels in A549 cells, indicating that this treatment inhibits angiogenesis. This finding is consistent with previous studies demonstrating the anti-angiogenic properties of Hesp in various cancer models [37]. By reducing VEGF expression, the combination therapy may limit the tumor’s ability to sustain growth and metastasize, further enhancing its therapeutic potential.

**Figure 8:**
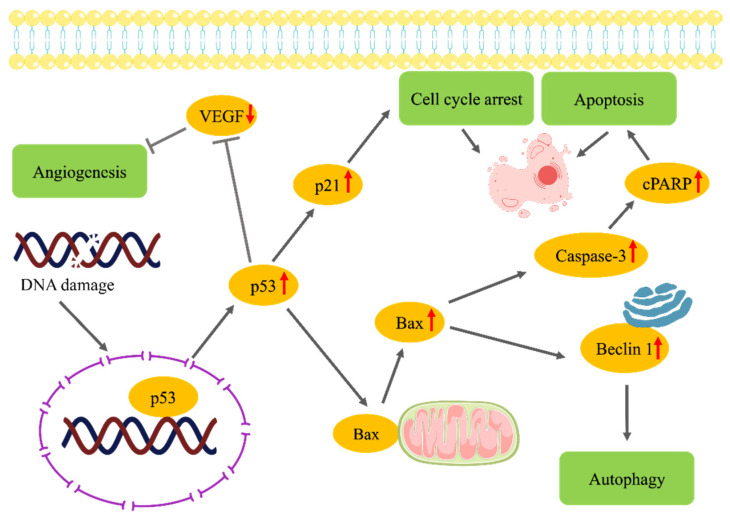
Molecular pathway mechanism of the potential effect of the combination of Hesp with BL on apoptosis, angiogenesis and autophagy in A549 lung cancer cells (upward and downward red arrows indicate the increase and decrease in protein expression rates induced by BL + Hesp combined treatment, respectively).

The use of phytochemicals such as Hesp in combination with traditional chemotherapeutic agents offers a promising approach to reducing the side effects of chemotherapy while enhancing efficacy. BL is known to cause significant toxicity, including pulmonary and gastrointestinal side effects, limiting its therapeutic window [38]. The addition of Hesp not only potentiates the anticancer effects of BL but also may allow for lower doses of BL to be used, reducing the likelihood of adverse effects. Importantly, Hesp has been shown to be safe in both preclinical and clinical settings, with no significant toxicity reported even at high doses [39,40]. These findings suggest that the combination of BL and Hesp could represent a more tolerable and effective treatment option for lung cancer patients. Although our study provides strong evidence of the synergistic effects of BL and Hesp on NSCLC cells, further research is needed to fully understand the underlying mechanisms of this interaction (Figure 8). In particular, in vivo studies are essential to assess the therapeutic potential and safety of this combination in a clinical setting. Moreover, it will be important to investigate whether the Hesp + BL combination is effective in other cancer types and to explore the potential for resistance mechanisms to develop over time.

## 4. Materials and Methods

### 4.1. Chemicals and Reagents

The chemicals and reagents used in the studies are as follows: bleomycin sulfate (MedChem, Monmouth Junction, NJ, USA); hesperidin and antibodies (Santa Cruz, Dallas, TX, USA); DMEM, penicillin/streptomycin, FBS, Trypsin-EDTA, DPBS (Gibco, NY, USA); trypan blue dye, TEMED, Tris, KCl, NaCl, HCl, NaF, Na_3_VO_4_, Commasie blue-G250, NaN_3_, luminol, skim milk powder, Ponceau S, H_3_PO_4_, p-coumaric acid, PMSF, DTT, benzamidine, ethanol, glycerin glycerol sodium dodecyl sulfate (Sigma-Aldrich, Burlington, MA, USA); Tris HCl, DMSO, Tween-20, Bromphenol blue, β-mercaptoethanol, NP-40, EDTA, EGTA,-glycerophosphate, H_2_O_2_, NaOH, glycine (Merck, Darmstadt, Germany); 0.45 µm PVDF membrane (Millipore, Darmstadt, Germany); 25 and 75 cm^2^ cell culture flasks (Sarstedt, Numbrecht, Germany); microcentrifuge tubes, 15 and 50 mL Eppendorf tubes (Isolab, Eschau, Germany); 6- and 96-well microplates (Corning, NY, USA); 5–10–25 mL sterile pipettes and plastic pipette tips, 3 and 6 cm Petri dishes (Costar, Washington, DC, USA). In addition, WST-1 Cell Proliferation and Cytotoxicity Test Kit (Boster, Pleasanton, CA, USA), Human Vascular Endothelial Cell Growth Factor A (VEGF-A) ELISA Kit (YL Biotech, Shanghai, China) and Caspase-3 Analysis Kit (BioVision, Milpitas, California, USA) were used in the studies according to the manufacturer’s protocols. All reagents, solvents and samples were prepared to be analytical grade and stored under appropriate conditions (Refrigerator 2–8 °C/SEG, Deep Freezer −20 °C/Vestel, Deep Freezer −86 °C/Nuaire Glacier NU-9668GC).

### 4.2. Cell Culture and Conditions

Human non-small cell lung cancer A549 cell line was used in the studies. A549 cells were obtained from Bingol University Cancer Research Laboratory. All studies were carried out under completely sterile conditions in a HEPA filter Biosafety Cabinet (Esco Micro Pte. Ltd., Singapore) with airflow. The cells were grown in high-glucose DMEM containing 10% FBS, 1% penicillin-streptomycin and 1% L-glutamine, and incubated in a CO_2_ incubator (Esco cell culture CO_2_ incubator, Singapore) at 37 °C, 5% CO_2_ and 95% humidity conditions. Cells were checked daily, and the medium was refreshed three times a week. When they reached 80% density, the cells were trypsinized and harvested for use in studies.

### 4.3. Cell Viability

The proliferation of A549 cells was determined by WST-1 cell viability analysis [9]. Briefly, after the cells were harvested, they were seeded into 96-well plates at 5 × 10^3^ cells/well and incubated overnight at 37 °C with 5% CO_2_. Cells were then exposed to either individual compounds or combinations. For single-agent treatments, Hesp or BL was administered at concentrations ranging from 0 to 100 µM for 24 h, 48 h or 72 h. For combination treatments, (i) BL was maintained at a constant concentration of 7.5 µM while Hesp was varied between 0 and 100 µM, or (ii) Hesp was held at a constant concentration of 12.5 µM while BL was increased within the same range. After treatment, 10 μL of WST-1 reagent was added to each well, the plates were incubated at 37 °C for additional 2 h, and absorbances were measured at 450 nm using a microplate reader (SpectraMax Plus 384, Molecular Devices, CA, USA). Percent proliferation was calculated by dividing the optical density of the treated group by the control group. The absorbance value of the mixture of DMEM and WST-1 reagent was used as background control. Using the viability values obtained, IC_50_ values for Hesp, BL and combination group were calculated.

### 4.4. Determination of Synergism by Combination Index (CI)

The combination index of Hesp and BL treatments in A549 cells was calculated by the following formula [19]:

CI = (A_BL_)_50_/(B_BL_)_50_ + (A_Hesp_)_50_/(B_Hesp_)_50_.

In this formula, (A_BL_)_50_ represents the concentration of BL that reduces cell viability by 50% when half the IC_50_ concentration of Hesp is applied, while (B_BL_)_50_ denotes the IC_50_ concentration of BL. Similarly, (A_Hesp_)_50_ indicates the concentration of Hesp that reduces cell viability by 50% when half the IC_50_ concentration of BL is applied, and (B_Hesp_)_50_ refers to the IC_50_ concentration of Hesp. The CI value is interpreted as follows: a CI greater than 1 indicates antagonism, a CI equal to 1 suggests an additive effect, and a CI less than 1 demonstrates synergism [18].

### 4.5. Clonogenic Assay

For each group, 5 × 10^2^ cells were planted in 6 cm Petri dishes and incubated at 37 °C with 5% CO_2_ overnight. A549 cells were then treated with BL (1 μM and 7.5 μM), Hesp (5 μM and 12.5 μM) and combinations (1 μM BL + 5 μΜ Hesp and 1 μM BL + 12.5 μΜ Hesp) for 72 h. After the procedure, the media were aspirated, and fresh, drug-free media were added to Petri dishes. Cells were kept in this medium for 12 days. At the end of this period, the media were aspirated, and the colonies formed were fixed with methanol/acetic acid (3:1/v:v) mixture for 5 min. This mixture was then removed, and the petri dishes were washed with cold PBS and stained with crystal violet (0.05%) for 15 min [20]. The colonies formed were counted and compared with the untreated group and percent colony formation values were calculated.

### 4.6. Wound-Healing Assay

In vitro wound-healing analysis was performed to determine the cell migration capacity of A549 cells [9]. A total of 3 × 10^5^ cells were seeded into each well of 6-well microplates, and after reaching a density of 80–90%, a linear scratch was made on the surface with a sterile pipette tip. The scraped cells were removed by washing with PBS. The wells were then treated with BL (7.5 μM), Hesp (12.5 μM) and combinations (7.5 μM BL + 12.5 μΜ Hesp) for 72 h. To assess the progress of wound healing, the scratch line was checked at 0, 6 and 24 h, and images were recorded with an inverted microscope (Olympus CKX 41). Quantification of wound closure was performed using the ImageJ 2.1 software. The initial wound area (at 0 h) and the remaining wound areas (at 6 and 24 h) were measured, and the percentage of wound closure was calculated using the following formula: percentage of wound closure = [(Initial wound area−Remaining wound area)/Initial wound area] × 100. This formula enabled precise normalization of wound-healing progression for each treatment group relative to the control.

### 4.7. Determination of VEGF Amount

For the quantification of VEGF, an important indicator of angiogenesis, the human vascular endothelial cell growth factor A (VEGF-A) ELISA Kit was used in accordance with the manufacturer’s protocols. Briefly, A549 cells were planted in 3 cm Petri dishes as 2 × 10^5^ cells/Petri dish and incubated overnight at 5% CO_2_ and 37 °C conditions. After 72 h of treatment, the cells were trypsinized and removed and transferred to sterile tubes. The tubes were then centrifuged at 2.500 rpm for 20 min and the supernatant fractions were removed. Amounts of 40 µL of supernatant, 10 µL of VEGF-A antibody and 50 µL of streptavidin-HRP were added to the wells of the 96-well plate. The cap of the microplate was sealed, and kept on a shaker at light intensity to mix the contents. It was then incubated at 37 °C for 60 min. At the end of this period, some of the liquid portion of the plates was removed, and the wash solution was added to the remaining portion and rinsed. After 30 s, the liquid part was removed, and 50 µL of chromogen solution A and B were added to the wells, respectively. The microplate was shaken and kept at 37 °C for 10 min in a non-light environment. Then, 50 µL of stop solution was added to each well and the transformation of the blue color to yellow was observed. Absorbance values were measured with a microplate reader (SpectraMax Plus 384, Molecular Devices, CA, USA) at 450 nm wavelength. The absorbance values of the standards included in the kit and prepared according to the protocol were normalized, and VEGF amounts were calculated in ng/L.

### 4.8. Morphological Investigation of Vacuolization

The neutral red staining method was used to examine autophagic vacuoles [23]. Briefly, control cells and treated cells (treated with 7.5 μM BL, 12.5 μM Hesp and combinations 7.5 μM BL + 12.5 μΜ Hesp for 72 h) were stained with neutral red dye at a concentration of 1/3000 (*w*/*v*) for 2–15 min and observed by microscope (Olympus CKX 41, Olympus Corp., Tokyo, Japan) after washing twice with PBS. Then, the total number of cells forming autophagic vacuoles and the number of normal cells were determined and the percentage of vacuolated cells was calculated.

### 4.9. Morphological Analysis of Apoptosis

Dual Acridine Orange (AO)/Ethidium Bromide (EB) staining was used to observe the apoptotic effects on cells morphologically [41]. After 72 h of treatment with 7.5 μM BL, 12.5 μM Hesp and 7.5 μM BL + 12.5 μΜ Hesp, AO/EB mixture was added to the wells at a concentration of 100 µg/mL. Morphological changes in the cells were then examined under an inverted microscope with a fluorescence attachment (Olympus CKX 41, Olympus Corp., Tokyo, Japan). At least one hundred cell counts were performed in triplicate, and apoptotic indices were calculated by determining the percentages of normal and apoptotic cells as a result of these counts.

### 4.10. Determination of Apoptosis by Caspase-3 ELISA

The effect of treatment groups on caspase-3 activity in A549 cells was quantitatively tested using the Caspase-3 Colorimetric Test Kit [42]. A total of 5 × 10^5^ cells were seeded into each well of 6-well microplates and left overnight at 37 °C. Later, the specified treatments were applied to cells except the control group for 72 h. The cells were then removed with trypsin and centrifuged to form pellets. For colorimetric analysis, these cells were suspended in chilled lysis buffer. The cell lysate was incubated on ice for 10 min before centrifugation and centrifuged at 10.000 x g in a refrigerated centrifuge (Universal 320R, Hettich) for 1 min. An amount of 50 µL of the supernatant was taken into a clean tube, and reaction buffer containing 10 mM dithiothreitol (DTT) was added and incubated on ice for 30 more min. Cell lysate (50 µL) and 50 µL reaction buffer mixture containing 10 mM DTT was transferred to wells of a 96-well microplate. An amount of 5 µL of 4 mM DEVD—pNA substrate was added to each well (to a final concentration of 200 µM), and then, it was incubated at 37 °C for 2 h. Absorbance values were recorded at 405 nm with the aid of a microplate reader (SpectraMax Plus 384, Molecular Devices). The values obtained were expressed in terms of fold change compared to the untreated control group.

### 4.11. Determination of Protein Expression Levels by Western Blot

For Western blot analysis, 5 × 10^5^ cells for each group were inoculated in 6 cm Petri dishes, and treatments were administered after an overnight incubation. Following a 24 h treatment period, cells were harvested and lysed on ice in cold RIPA buffer containing protease and phosphatase inhibitors. The total amount of protein was determined by the Bradford method [43]. Then, protein from each group was separated by 10–12% SDS-PAGE according to the density, and the protein bands were transferred to a polyvinylidene difluoride (PVDF) membrane. Membranes were blocked with 5% skim milk in Tris buffered saline containing 1% Tween 20 (TBS-T, pH 7.4) for 1 h at room temperature and incubated with primary antibody 1: 1000 at 4 °C overnight. Primary and secondary antibodies used in Western blot studies and their dilution rates are shown in Table 1.

Each of the membranes was washed five times with TBS-T for 10 min at room temperature and incubated again with HRP-conjugated secondary antibody (1: 1.000) for 3 h at room temperature. Membranes were then rewashed five times with TBS-T. Then, 4 mL each of ECL stock solution 1 in membranes (0.5 mL of 250 mM luminol with 0.22 mL of 90 mM p-coumaric acid and 5 mL of 1 M Tris (pH 8.5); total volume 50 mL) and ECL stock solution 2 (31 µL of 30% H_2_O_2_ and 5 mL of 1 M Tris (pH 8.5; total volume 50 mL)) was placed in the container and shaken in these solutions for 15 s. Then, the membrane was placed in the cassette, and the tape images on the membranes were transferred to X-Ray films with an imaging device (Carestream Medical X-Ray Processor, Atlanta, GA, USA). Bands were analyzed quantitatively using ImageJ software version 2.1. Densitometrical readings of the bands were normalized according to tubulin expression [44,45].

### 4.12. Statistical Analysis

All results are expressed as mean ± standard deviation (SD) of at least three experiments. One-way ANOVA and Tukey’s multiple comparison test were used to compare treatment groups with each other and with controls. GraphPad Prism 8.0 and Windows Office-Excel programs were used for statistical analysis. Statistical significance was accepted as *p* < 0.05.

## 5. Conclusions

This study demonstrates that the combination of Hesp with BL significantly enhances the antiproliferative, apoptotic, autophagic, and anti-angiogenic effects in A549 cells. The combination therapy led to a substantial increase in key apoptotic markers such as p53, p21, Bax, and cPARP, indicating a robust activation of apoptosis, particularly through the intrinsic mitochondrial pathway. Additionally, the upregulation of Beclin-1 suggests that autophagy was also induced, potentially contributing to the overall cell death process. The observed reduction in VEGF levels further highlights the anti-angiogenic potential of this treatment, which could help inhibit tumor growth and metastasis. The synergistic effects of Hesp and BL suggest that combining traditional chemotherapy with natural compounds can improve therapeutic efficacy while potentially reducing the side effects associated with high-dose chemotherapy. Given the low toxicity and safety profile of Hesp, this combination represents a promising therapeutic strategy for the treatment of lung cancer and possibly other cancers. The primary limitation of this study is its focus on a single lung cancer cell line. Conducting future research using a broader range of lung cancer cell lines will be crucial to reinforcing these conclusions. Nonetheless, the combination of Hesp with BL presents a promising strategy that could improve treatment outcomes while offering a more tolerable therapeutic option for lung cancer patients.

## Figures and Tables

**Table 1:** Primary and secondary antibodies used in Western blot analysis.

Primary Antibody	Dilution Ratio	Secondary Antibody	Dilution Ratio
Beclin-1	1:1000	Goat anti rabbit IG-g HRP	1:1000
p53	1:1000	Goat anti mouse IG-g HRP	1:1000
p21	1:1000	Goat anti rabbit IG-g HRP	1:1000
Bax	1:1000	Goat anti rabbit IG-g HRP	1:1000
cPARP	1:1000	Goat anti rabbit IG-g HRP	1:1000
Tubulin	1:1000	Goat anti mouse IG-g HRP	1:1000

## Data Availability

The datasets generated and analyzed during the current study are fully presented in the manuscript, including tables and figures.
